# Supplementary material for: Pof8 is a La-related protein and a constitutive component of telomerase in fission yeast
Source: Nat Commun. 2018 Feb 8;9:587. doi: 10.1038/s41467-017-02284-8 (PMC5805746; doi:10.1038/s41467-017-02284-8)
Supplement: Supplementary file 1 — Supplementary Information(PDF 86303 kb) [file 41467_2017_2284_MOESM1_ESM.pdf]

The Supplementary Information file initially published online was corrupted and was replaced on 21/02/18.

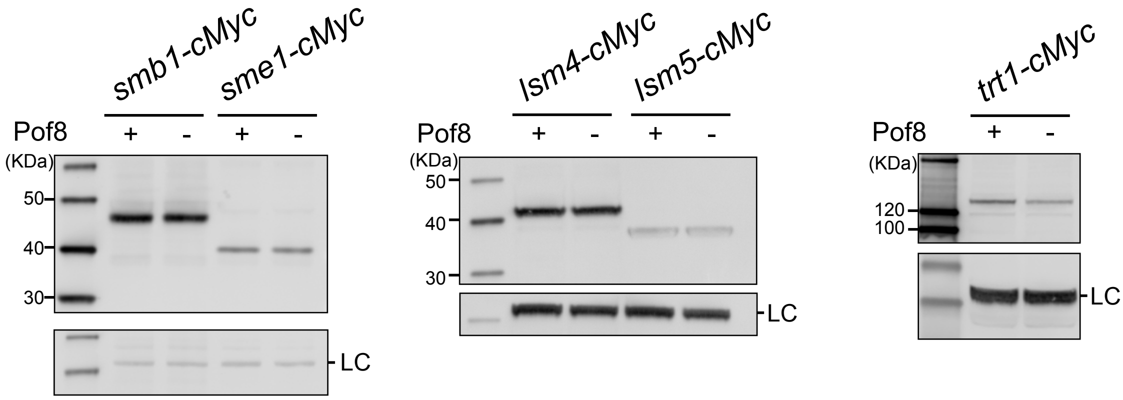

Supplementary Figure 1: Effect of pof8 deletion on protein levels of Sm, Lsm and Trt1. Cell-free extracts from strains with c-Myc epitope tags on the indicated proteins were subjected to Western analysis. For Lsm4, Lsm5 and Trt1, an antibody against α-tubulin was used as loading control; for Sm proteins, a non-specific band recognized by α-cMyc was used as a loading control.

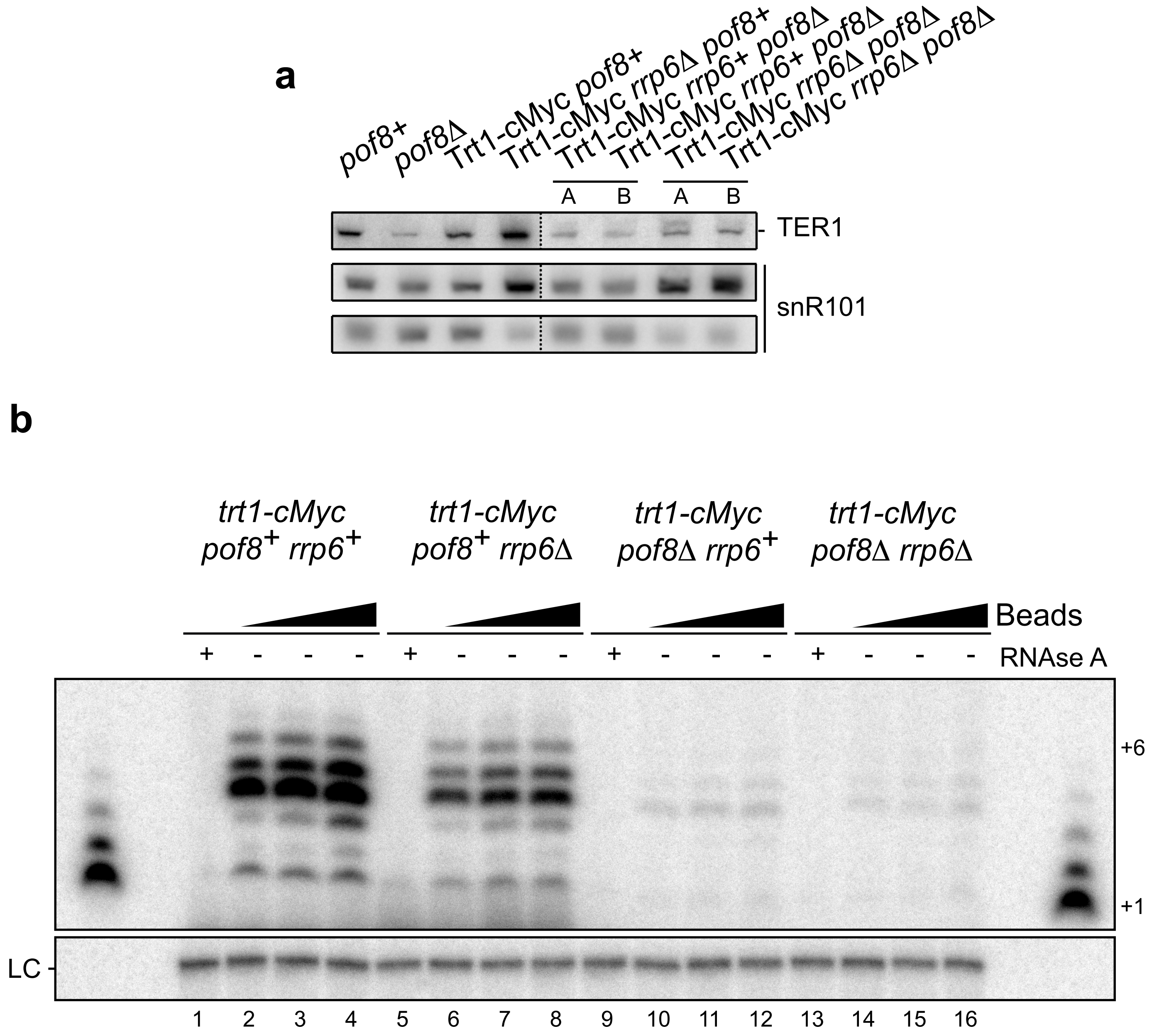

Supplementary Figure 2: Compromising TER1 degradation does not rescue pof8∆ effect on telomerase activity. (a) Northern for TER1 on total RNA samples from strains of the indicated genotypes. Both bands corresponding to snRNA101 are shown revealing a role for Rrp6 in the processing of the longer form. **(b)** Telomerase activity assay from pof8∆ and rrp6∆ strains.

a

Figure 2a

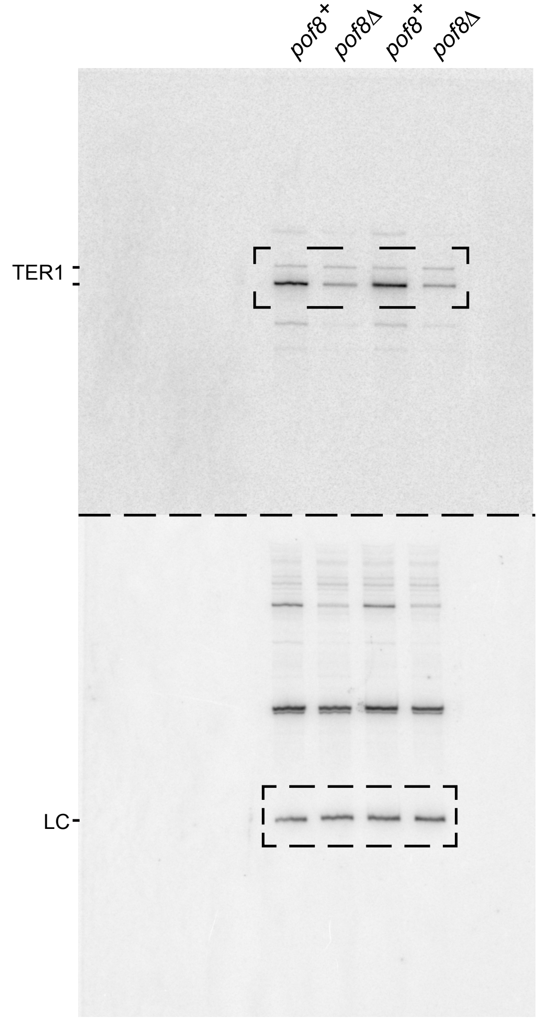

Figure 2c

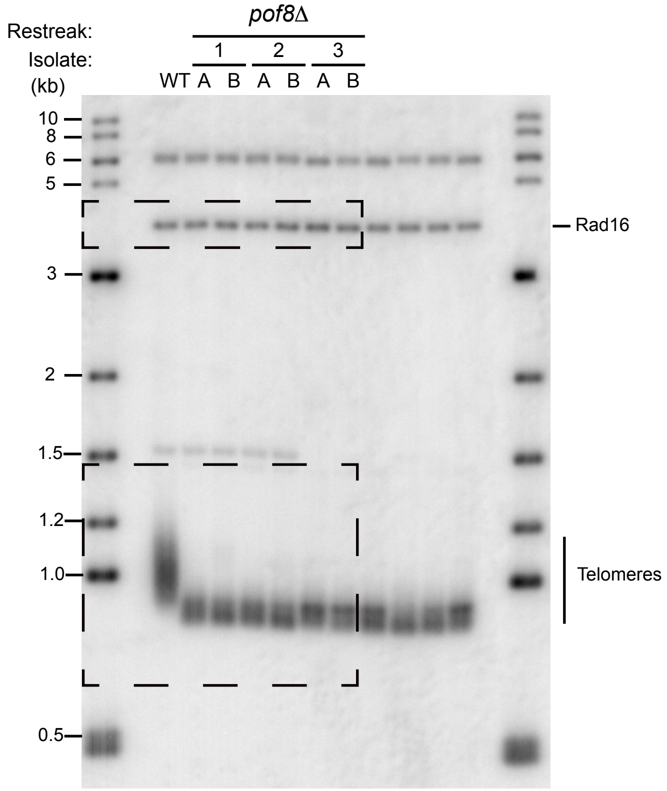

Figure 2e

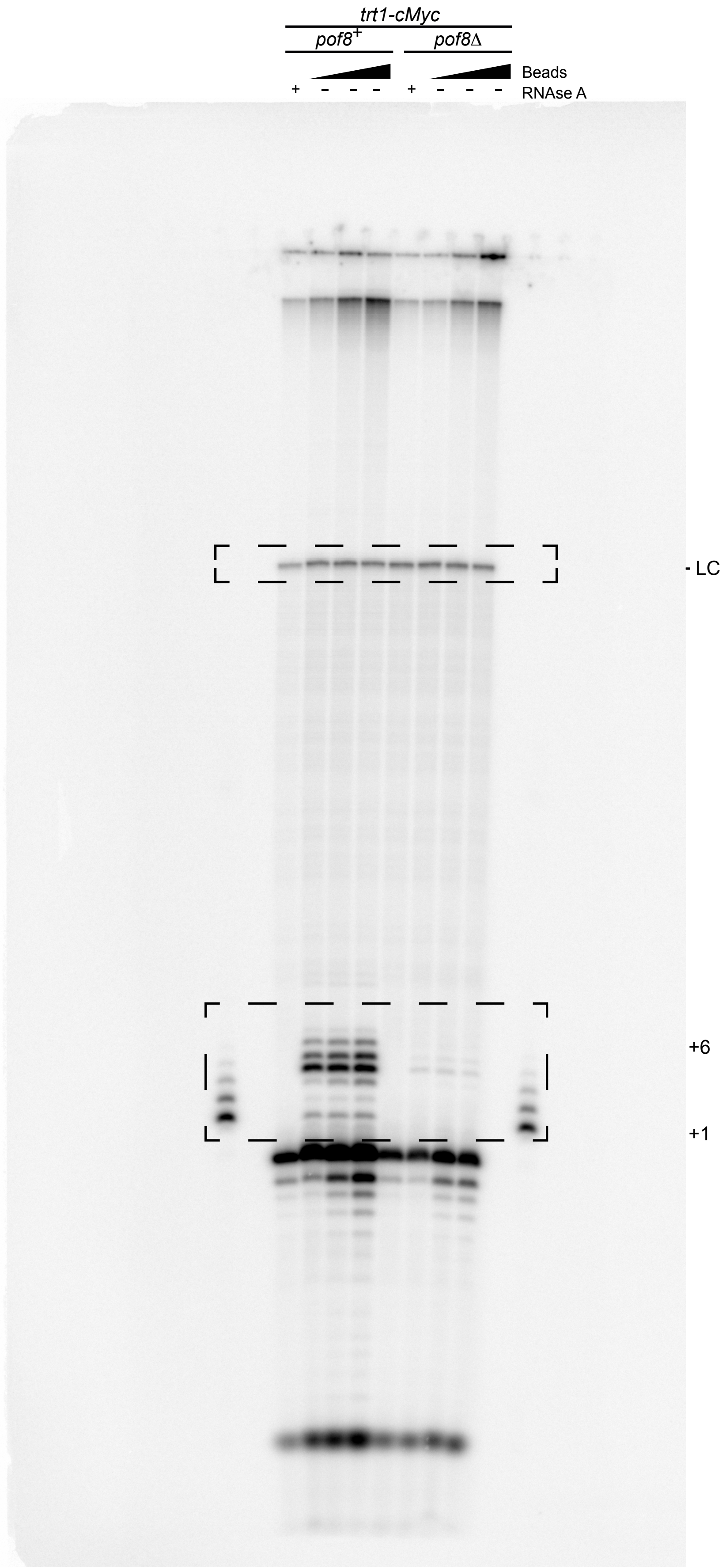

Paez-Moscoso et al.; Supplementary Figure 3

b

Figure 3a

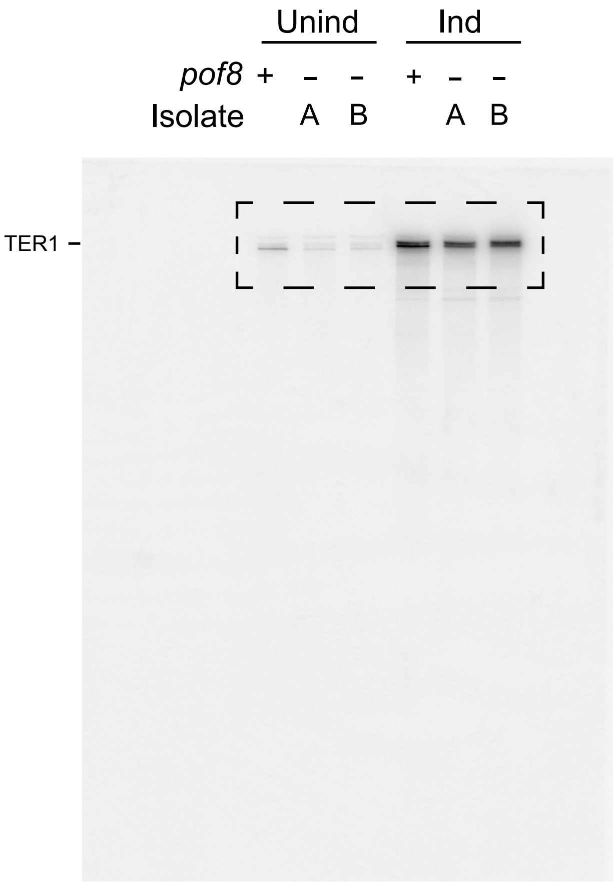

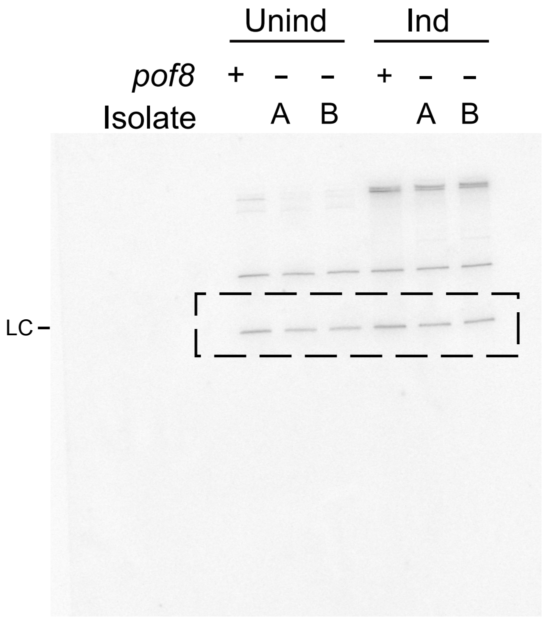

Figure 3b

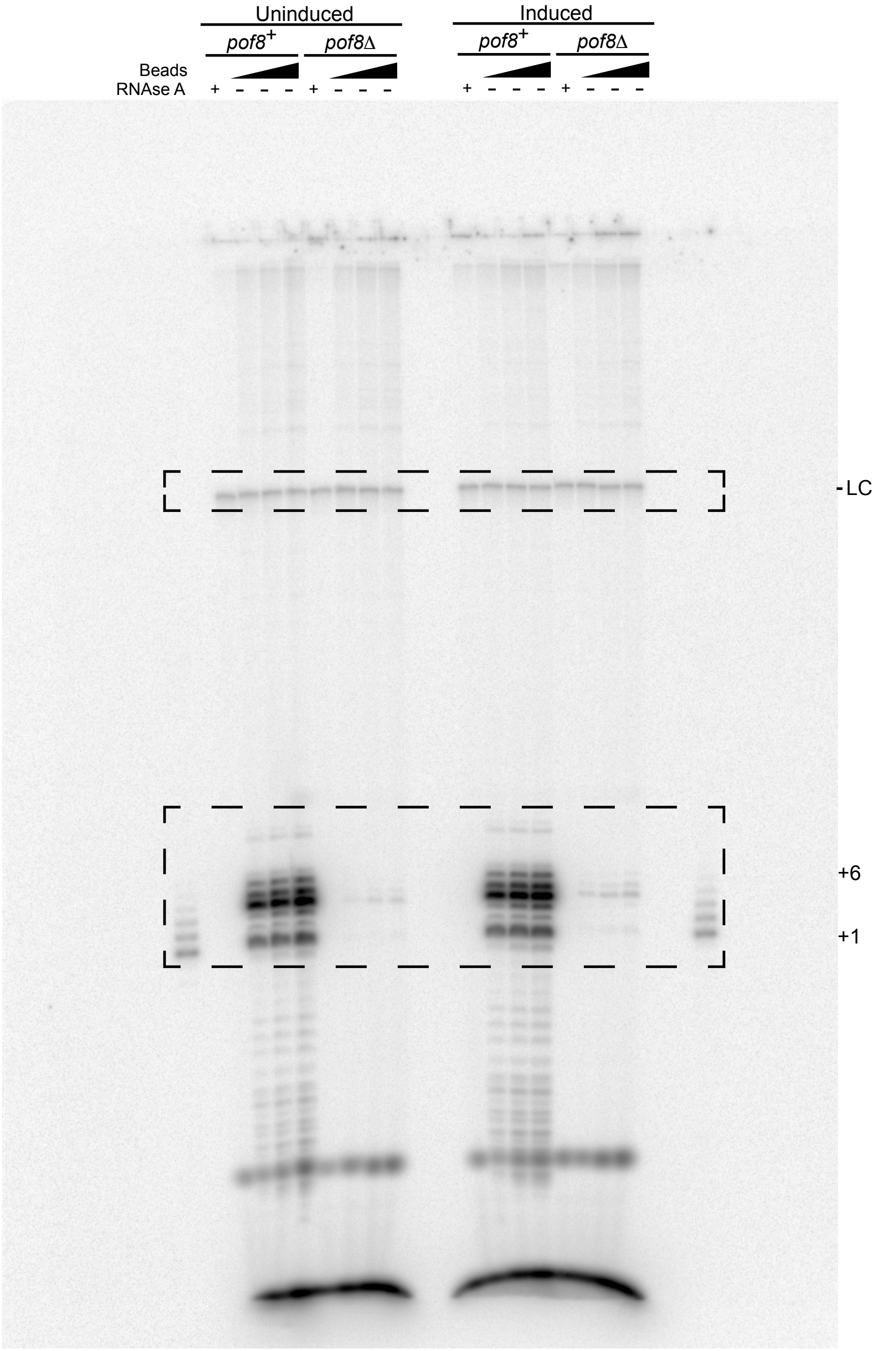

Paez-Moscoso et al.; Supplementary Figure 3

c

Figure 4a

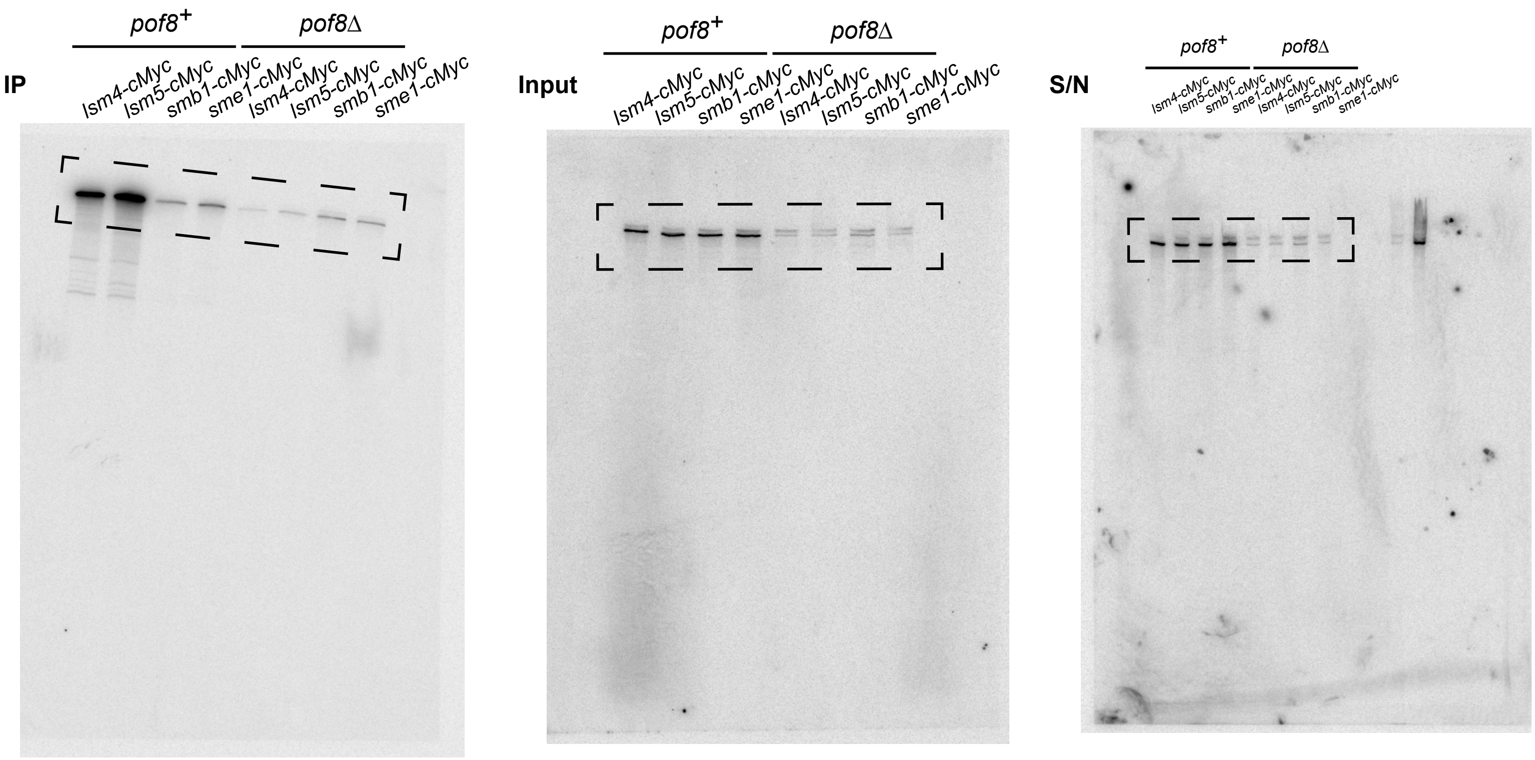

Figure 4b

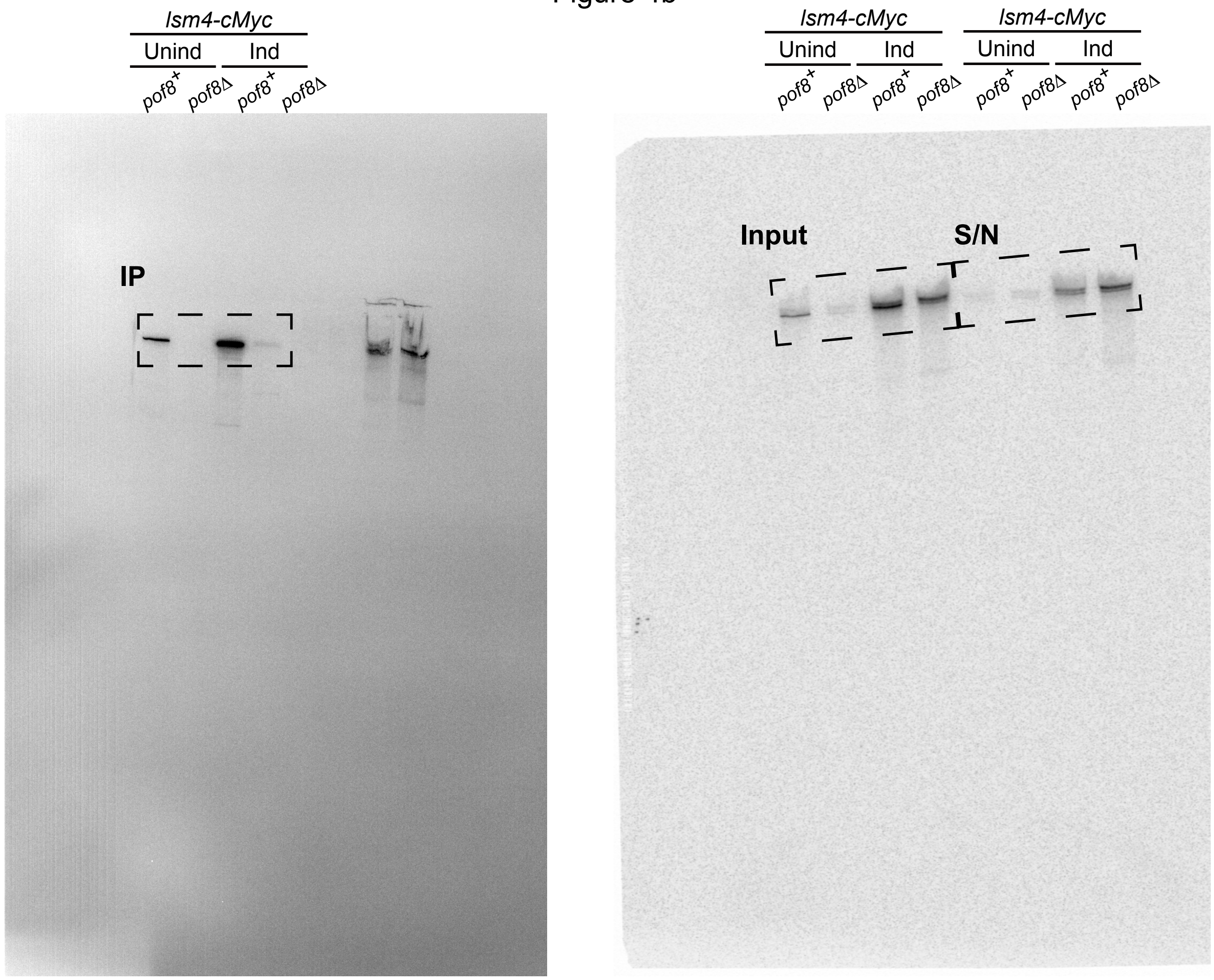

Paez-Moscoso et al.; Supplementary Figure 3

d

Figure 6a

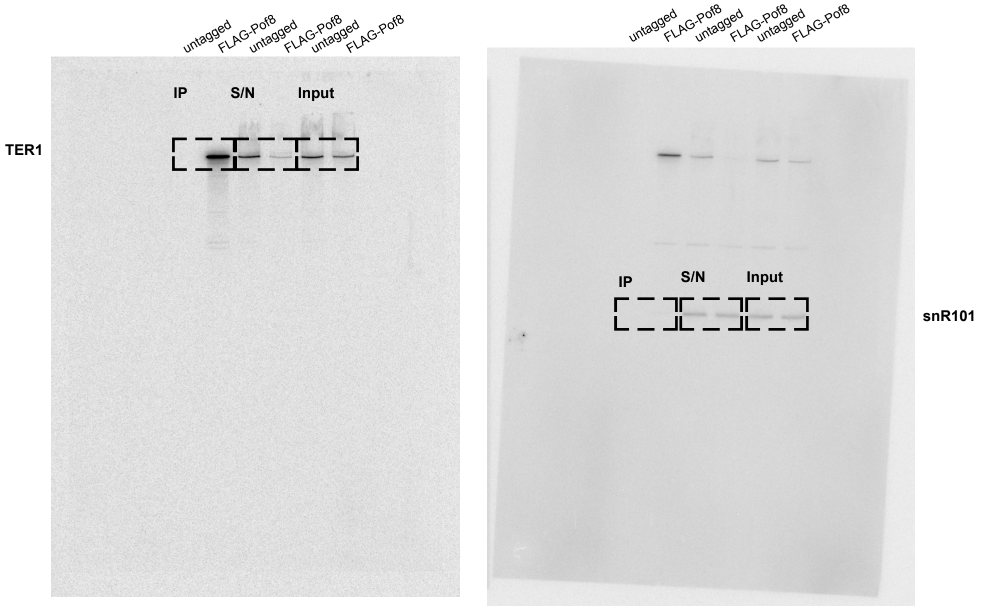

Figure 6c

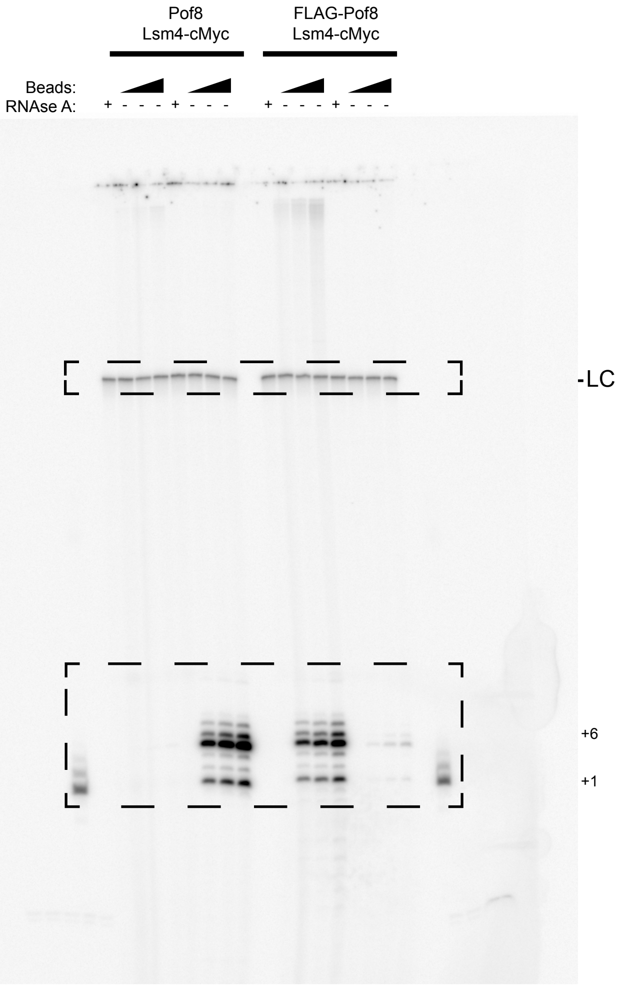

Supplementary Figure 3: Complete scans of blots presented in main figures as indicated. **(a)** Uncropped blots from Figure 2a, c, e. **(b)** Uncropped blots from Figure 3a and b. **(c)** Uncropped blots from Figure 4a and b. **(d)** Uncropped blots from Figure 6a and c.

Supplementary Table 1: *S. pombe* strains used in this study

Strain name	Genotype	Source	Figure
PP138	h^- *ade6-M216 leu1-32 ura4-D18 his3-D1*	*Lab stock*	2a, 2b, 2c, 2d, 5c, 7b, S2a
PP298	h^- *ade6-M210 leu1-32 ura4-D18 his3-D1 trt1:: trt1-Cmyc*$_9$	*Supp. Ref*[1]	2e, S1, S2a, S2b
PP407	$h^{+/-}$ *ade6-M210/ade6-M216 leu1-32/ leu1-32 ura4-D18/ ura4-D18 his3-D1/ his3-D1 ter1*$^+$*/ter1::kanMX6*	*Supp. Ref*[2]	2d
PP577	h^- *ade6-M216 leu1-32 ura4-D18 his3-D1 lsm4::lsm4-myc*$_{13}$*-natMX6*	*Ref*[8]	2f, 4a, 5b, S1
PP578	h^- *ade6-M216 leu1-32 ura4-D18 his3-D1 lsm5::lsm5-myc*$_{13}$*-natMX6*	*Ref*[8]	2f, 4a, S1
PP580	h^- *ade6-M216 leu1-32 ura4-D18 his3-D1 smb1:: smb1-myc*$_{13}$*-natMX6*	*Ref*[8]	4a, S1
PP582	h^- *ade6-M216 leu1-32 ura4-D18 his3-D1 sme1::sme1-myc*$_{13}$*-natMX6*	*Ref*[8]	4a, S1
PP585	h^- *ade6-M216 leu1-32 ura4-D18 his3-D1 lsm8::lsm8-myc*$_{13}$*-natMX6*	*Ref*[8]	7b
PP769	$h^?$ *ade6-M? leu1-32 ura4? his3? trt1::trt1-Cmyc*$_9$ *rrp6::kanMX6*	*This study*	S2a, S2b
PP1721	$h^?$ *ade6-M? leu1-32 ura4-D18 his3-D1 lsm4::lsm4-myc*$_{13}$*-natMX6 aur1::[pCST159 -ter1]*	*This study*	3a, 3b, 3c, 4b
PP1723	h^- *ade6-M216 leu1-32 ura4-D18 his3-D1 pof8::kanMX6*	*This study*	2a, 2b, 2c, 2d, 7b, S2a
PP1724	h^- *ade6-M216 leu1-32 ura4-D18 his3-D1 lsm4::lsm4-myc*$_{13}$*-natMX6 pof8::kanMX6*	*This study*	2f, 4a, 5b, S1
PP1725	h^- *ade6-M216 leu1-32 ura4-D18 his3-D1 lsm5::lsm5-myc*$_{13}$*-natMX6 pof8::kanMX6*	*This study*	2f, 4a, S1
PP1726	h^- *ade6-M216 leu1-32 ura4-D18 his3-D1 smb1::smb1-myc*$_{13}$*-natMX6 pof8::kanMX6*	*This study*	4a, S1

PP1727	*h^- ade6-M216 leu1-32 ura4-D18 his3-D1 sme1::sme1-myc_{13}-nat, pof8::kanMX6*	*This study*	4a, S1
PP1728	*h^- ade6-M210 leu1-32 ura4-D18 his3-D1 trt1::trt1-$Cmyc_9$ pof8::kanMX6*	*This study*	2e, S1, S2a, S2b
PP1729	*$h^?$ ade6-M210 leu1-32 ura4-D18 his3-D1 trt1::trt1-$Cmyc_9$ pof8::kanMX6 rrp6::nat*	*This study*	S2a, S2b
PP1797	*$h^?$ ade6-M? leu1-32 ura4-D18 his3-D1 lsm4::lsm4-myc_{13}-nat aur1::[pCST159-ter1] pof8::kanMX6*	*This study*	3a, 3b, 3c, 4b
FP1546	*h- ade6-M216 leu1-32 ura4-D18 his3-D1 lsm4::lsm4-myc13-natMX6 pof8::kanMX6 [pDBlet-Pof8]*	*This study*	5a, 5b, 6a, 6b, 6c, 7a
FP1547	*h- ade6-M216 leu1-32 ura4-D18 his3-D1 lsm4:: lsm4-myc13-natMX6 pof8::kanMX6 [pDBlet-3xFLAG-Pof8]*	*This study*	5a, 5b, 5c, 6a, 6b, 6c, 7a

References

1. Haering, C.H., Nakamura, T.M., Baumann, P. & Cech, T.R. Analysis of telomerase catalytic subunit mutants in vivo and in vitro in Schizosaccharomycespombe. *Proc Natl Acad Sci U S A* **97**, 6367-72 (2000).
2. Box, J.A., Bunch, J.T., Tang, W. & Baumann, P. Spliceosomal cleavage generates the 3' end of telomerase RNA. *Nature* **456**, 910-4 (2008).

Supplementary Table 2: Oligonucleotides used to generate deletion, fusion and integration constructs

Product description	Primer #	Sequence
kanMX6 cassette	BLoli6138/<br>BLoli6139	5′-GCGAAGTAAACGGATCCCCGGGTTAATTAAG-3'/<br>5′-GGAAAACATAGAATTCGAGCTCGTTTAAACTG-3'
Pof8 5' UTR	BLoli6136/<br>BLoli6137	5′-TCTAAAGTTCGTCTTTTGCATAAC-3'/<br>5′-CGGGGATCCGTTTACTTCGCTCCTTAAAGTAC-3'
Pof8 3' UTR	BLoli6140/<br>BLoli6141	5′-GCTCGAATTCTATGTTTTCCTTTCTCTGGTAATAC-3'/<br>5′-GCTTTCTTATTTGTAGAGACAATTG-3'
Pof8::kanMX6	BLoli6136/<br>BLoli6141	5′-TCTAAAGTTCGTCTTTTGCATAAC-3'/<br>5′-GCTTTCTTATTTGTAGAGACAATTG-3'
cloning of Pof8 into pDBlet	BLoli6676/<br>BLoli6677	5′-AAAA GAATTC AACATGGCAACTGCGACCAA-3'/<br>5′-CGATAAGCTT CTTCCAATAGCTCGGTTTGT-3'
3xFLAG-Pof8 5' UTR in pDBlet	BLoli6676/<br>BLoli6683	5′-AAAA GAATTC AACATGGCAACTGCGACCAA-3'/<br>5′-ATCGTGATCTTTGTAGTCCATTTTACTTCGCTCCTTAAAG-3'
3xFLAG-Pof8 in pDBlet	BLol6684/<br>BLoli6677	5′-AAGTAAAATGGACTACAAAGATCACGATGGAGATTATAAAGACCATGATATAGATTATAAGGATGACGATGACAAGTTTGTGCCAAGGCAACTG-3'/<br>5′-CGATAAGCTT CTTCCAATAGCTCGGTTTGT-3'
3xFLAG-Pof8 into pDBlet	BLoli6676/<br>BLoli6677	5′-AAAA GAATTC AACATGGCAACTGCGACCAA-3'/<br>5′-CGATAAGCTT CTTCCAATAGCTCGGTTTGT-3'
Sp6-Ter1 5' (+1 to +97)	BLoli7098/<br>BLoli7099	5'-TACGATTTAGGTGACACTATAGATACTCAACGCAACGCC-3'/<br>5'-CAAGGAAATTATTTCCTTCAAACTTTCAAATCAATCAC-3'
3' arm Ter1 (+955 to +1212)-HDV	BLoli7100/<br>BLoli6540	5'-TGAAGGAAATAATTTCCTTGAACTTGGATTCTTTG-3'/<br>5'-TTGGTCCCATTCGCCATGC-3'
Sp6-Ter1-HDV probe	BLoli7098/<br>BLoli6540	5'-TACGATTTAGGTGACACTATAGATACTCAACGCAACGCC-3'/<br>5'-TTGGTCCCATTCGCCATGC-3'

Supplementary Table 3: Genes affected in expression level by the deletion of *pof8*
(a) Genes with RNA levels decreased by more than 2-fold in the absence of Pof8 based on an EdgeR analysis of triplicate *pof8⁺* and *pof8Δ* RNA samples. **(b)** Genes with increased expression by more than two-fold in the same dataset as in (a).

a

gene ID	external gene ID	log FC	adj. p value	gene biotype
SPAC17G6.17	pof8	-3.1722	2.27E-44	protein_coding
SPNCRNA.214	ter1	-2.0020	6.76E-54	ncRNA
SPNCRNA.1554	SPNCRNA.1554	-1.9279	1.62E-13	ncRNA
SPAC29A4.12c	mug108	-1.8857	3.72E-17	protein_coding
SPNCRNA.690	prh1-antisense-1	-1.7065	2.58E-15	ncRNA
SPNCRNA.1340	SPNCRNA.1340	-1.6384	2.00E-20	ncRNA
SPNCRNA.716	SPAC56F8.12-antisense-1	-1.3851	5.08E-41	ncRNA
SPNCRNA.742	SPAC9.08c-antisense-1	-1.2879	6.18E-16	ncRNA
SPNCRNA.322	SPNCRNA.322	-1.2691	3.72E-17	ncRNA
SPCC569.02c	SPCC569.02c	-1.2528	1.34E-08	protein_coding
SPNCRNA.784	SPNCRNA.784	-1.2273	1.10E-06	ncRNA
SPNCRNA.1424	SPNCRNA.1424	-1.2010	1.37E-09	ncRNA
SPNCRNA.794	SPNCRNA.794	-1.1924	2.28E-08	ncRNA
SPBPB21E7.01c	eno102	-1.1252	2.39E-05	protein_coding
SPAPB24D3.07c	SPAPB24D3.07c	-1.0590	1.78E-28	protein_coding
SPBPB2B2.01	SPBPB2B2.01	-1.0412	3.06E-05	protein_coding
SPNCRNA.888	end4-antisense-1	-1.0053	1.41E-07	ncRNA
SPNCRNA.648	SPNCRNA.648	-1.0025	1.40E-07	ncRNA
SPNCRNA.779	SPNCRNA.779	-1.0016	5.71E-05	ncRNA

b

gene ID	external gene ID	log FC	adj. p value	gene biotype
SPCC1020.09	gnr1	1.5878	2.20E-12	protein_coding
SPBCPT2R1.08c	tlh2	1.3332	5.09E-11	protein_coding
SPAC27D7.11c	SPAC27D7.11c	1.2919	1.39E-43	protein_coding
SPBC839.06	cta3	1.2484	8.90E-09	protein_coding
SPRRNA.02	15S_rRNA	1.1726	3.69E-02	rRNA
SPBC685.07c	rpl2701	1.1687	2.69E-20	protein_coding
SPAC13G7.08c	crb3	1.1250	1.49E-16	protein_coding
SPCC162.05	coq3	1.0501	4.39E-05	protein_coding
SPRRNA.01	21S_rRNA	1.0212	3.19E-02	rRNA

Supplementary Table 4: Genes enriched in Lsm8-cMyc immunoprecipitation

gene ID	external gene ID	control normalized read count	Lsm8 IP normalized read count	FC	gene_biotype	comment
SPNCRNA.1532	SPNCRNA.1532	0.8	742.6	915.84	ncRNA	annotation overlaps with 1531
SPNCRNA.1531	SPNCRNA.1531	0.9	743.0	785.41	ncRNA	annotation overlaps with 1532
SPSNRNA.04	snu4	78.1	36400.4	466.19	snRNA	
SPNCRNA.214	ter1	3.4	534.1	159.38	ncRNA	
SPNCRNA.240	SPNCRNA.240	0.7	96.1	142.30	ncRNA	
SPSNORNA.39	snR36	0.4	45.8	112.88	snoRNA	
SPBC16E9.18	psd1	1.3	45.9	34.68	protein_coding	
SPSNRNA.06	snu6	387.3	11785.5	30.43	snRNA	
SPBTRNAGLY.09	SPBTRNAGLY.09	0.7	18.7	27.70	tRNA	
SPNCRNA.895	cox15-antisense-1	1.1	27.4	26.02	ncRNA	
SPAC1486.08	cox16	1.2	28.1	23.13	protein_coding	
SPSNRNA.02	snu2	12.2	227.9	18.74	snRNA	
SPSNORNA.21	snoU14	3.8	70.5	18.38	snoRNA	
SPAC24C9.16c	cox8	1.2	20.3	17.05	protein_coding	
SPNCRNA.445	snoR61	2.2	26.9	12.46	snoRNA	
SPSNRNA.05	snu5	8.7	108.3	12.40	snRNA	
SPAC10F6.17c	SPAC10F6.17c	1.6	13.6	8.69	protein_coding	
SPAC1B3.12c	rpb10	4.1	31.2	7.64	protein_coding	
SPBC354.06	mrps16	1.8	12.1	6.61	protein_coding	
SPNCRNA.905	SPAC8E11.01c-antisense-1	2.5	15.7	6.37	ncRNA	
SPMIT.07	atp6	5.5	31.3	5.73	protein_coding	
SPAC3H5.05c	rps1401	10.8	53.0	4.93	protein_coding	
SPBC1539.06	acb1	3.2	15.0	4.75	protein_coding	
SPAC10F6.16	mug134	3.2	15.4	4.75	protein_coding	
SPAC3H5.04	aar2	11.8	53.3	4.52	protein_coding	
SPAC4F10.20	grx1	4.3	17.5	4.04	protein_coding	
SPAPB15E9.01c	pfl2	10.7	42.3	3.95	protein_coding	
SPSNORNA.40	snR42	12.6	41.0	3.25	snoRNA	
SPSNORNA.32	sno12	34.1	103.4	3.04	snoRNA	
SPNCRNA.1464	SPNCRNA.1464	8.4	23.0	2.73	ncRNA	
SPAC24C9.03	mvd1	9.6	24.7	2.58	protein_coding	
SPAC343.12	rds1	7.9	20.1	2.53	protein_coding	
SPAC1635.01	SPAC1635.01	8.8	21.4	2.43	protein_coding	
SPAC343.20	SPAC343.20	8.0	19.2	2.40	protein_coding	
SPBC8D2.04	hht2	9.3	21.0	2.25	protein_coding	
